# Metal (Ag/Ti)-Containing Hydrogenated Amorphous Carbon Nanocomposite Films with Enhanced Nanoscratch Resistance: Hybrid PECVD/PVD System and Microstructural Characteristics

**DOI:** 10.3390/nano8040209

**Published:** 2018-03-30

**Authors:** Marios Constantinou, Petros Nikolaou, Loukas Koutsokeras, Apostolos Avgeropoulos, Dimitrios Moschovas, Constantinos Varotsis, Panos Patsalas, Pantelis Kelires, Georgios Constantinides

**Affiliations:** 1 Research Unit for Nanostructured Materials Systems, Department of Mechanical Engineering and Materials Science and Engineering, Cyprus University of Technology, 3041 Lemesos, Cyprus; m.k.constantinou@cut.ac.cy (M.C.); petros.nikolaou@cut.ac.cy (P.N.); l.koutsokeras@cut.ac.cy (L.K.); pantelis.kelires@cut.ac.cy (P.K.); 2 Department of Materials Science and Engineering, University of Ioannina, University Campus, 45110 Ioannina, Greece; aavger@cc.uoi.gr (A.A.); dmoschov@cc.uoi.gr (D.M.); 3 Department of Environmental Science and Technology, Cyprus University of Technology, 3041 Lemesos, Cyprus; c.varotsis@cut.ac.cy; 4 Department of Physics, Aristotle University of Thessaloniki, 54124 Thessaloniki, Greece; ppats@physics.auth.gr

**Keywords:** hydrogenated amorphous carbon films, metallic nanoparticles, hybrid deposition system, nanoscratch, nanocomposites

## Abstract

This study aimed to develop hydrogenated amorphous carbon thin films with embedded metallic nanoparticles (a–C:H:Me) of controlled size and concentration. Towards this end, a novel hybrid deposition system is presented that uses a combination of Plasma Enhanced Chemical Vapor Deposition (PECVD) and Physical Vapor Deposition (PVD) technologies. The a–C:H matrix was deposited through the acceleration of carbon ions generated through a radio-frequency (RF) plasma source by cracking methane, whereas metallic nanoparticles were generated and deposited using terminated gas condensation (TGC) technology. The resulting material was a hydrogenated amorphous carbon film with controlled physical properties and evenly dispersed metallic nanoparticles (here Ag or Ti). The physical, chemical, morphological and mechanical characteristics of the films were investigated through X-ray reflectivity (XRR), Raman spectroscopy, Scanning Electron Microscopy (SEM), Atomic Force Microscopy (AFM), Transmission Electron Microscopy (TEM) and nanoscratch testing. The resulting amorphous carbon metal nanocomposite films (a–C:H:Ag and a–C:H:Ti) exhibited enhanced nanoscratch resistance (up to +50%) and low values of friction coefficient (<0.05), properties desirable for protective coatings and/or solid lubricant applications. The ability to form nanocomposite structures with tunable coating performance by potentially controlling the carbon bonding, hydrogen content, and the type/size/percent of metallic nanoparticles opens new avenues for a broad range of applications in which mechanical, physical, biological and/or combinatorial properties are required.

## 1. Introduction

Hydrogenated amorphous carbon (a–C:H) thin films consist of carbon and hydrogen elements, where carbon atoms form dangling bonds with hydrogen or sp^1^, sp^2^, and sp^3^ bonds with other two, three or four carbon atoms, respectively. The properties of such films are directly linked to the hydrogen content and the hybridization state of carbon bonds. In general, high sp^2^ content promotes graphite-like properties, whereas high sp^3^ percentages favor diamond-like characteristics. The sp^2^ structure is relatively softer, more compliant and with lower coefficient of friction compared to the sp^3^ structure that is stiff and hard but brittle. The introduction of hydrogen tends to soften the material, lower its coefficient of friction and increase its ductility [1]. Amorphous carbon films (hydrogenated or not) with a significant percentage of sp^3^ hybridization, commonly referred to as diamond like carbon or DLC/DLCH in short, possess excellent properties like high stiffness and hardness, low coefficient of friction, high wear resistance, chemical inertness, low permeability, high melting point, optical transparency in infra-red (IR), high electrical resistivity and thermal conductivity [2,3,4]. These exceptional properties led to a series of industrial applications like protective coatings on microelectromechanical systems (MEMS), razor blades, hard disc drives and biomedical implants to gas barriers in polyethylene terephthalate (PET) bottles [5,6,7].

The main hindrance to further industrial exploitation of DLC films was poor scratch resistance which led to premature failure, primarily due to the high residual compressive stresses (in the GPa range) trapped during deposition and the ion subplantation process [8]. To circumvent this brittleness problem and allow for thicker and more stable films several recent studies proposed the doping of DLC with other elements [9,10,11,12,13]. Among the doping elements used were transition metals–typically termed metal containing hydrogenated amorphous carbon films, a–C:H:Me—or non-metal elements–typically termed as modified hydrogenated amorphous carbon films, a–C:H:X—such as silicon, oxygen, boron, nitrogen, and so forth. In essence, the idea lies in the formation of a particle reinforced nanostructured nanocomposite film for which the amorphous carbon matrix phase surrounds the soft metallic phase. For such composites, the toughening mechanism is based on continuum mechanics, where the cumulative composite response relates to the properties of the constituent phases, their relative fractions, the characteristic geometry of the dispersed phase (i.e., size, shape, dispersion and orientation) and finally the very nature of the bond between the matrix and the particulate nanoconstituents. Note that for thin films on substrate material the interfacial nature and residual stresses are also of important consideration. While there are other routes to improve the toughness of amorphous carbon films, like modifying the chemical structure, bonding characteristics or morphological details of the a–C matrix [7,14], we here concentrate on incorporating metallic nanoparticles into the hydrogenated amorphous carbon matrix.

The motivation of this study was the lack of (a) a concentrated study for the in-depth understanding of the structural and mechanical characteristics of a–C:H:Me and (b) deposition methodology in which the size of the reinforcing particles can be separately controlled and not be the result of the ion interaction with the substrate material. We aimed to develop a new generation of a–C:H:Ag and a–C:H:Ti films with improved combination of properties and performance. These characteristics have been achieved through the use of a hybrid deposition technology, a combination of Plasma Enhanced Chemical Vapor Deposition (PECVD) and Physical Vapor Deposition (PVD). The amorphous carbon matrix was generated by PECVD where carbon ions were generated by an RF plasma source by cracking methane, while the metallic nanoparticles (NPs) were generated through a nanoparticle source based on PVD technology. The motivation of using the above deposition system was associated with the flexibility of individually controlling the matrix quality and nanoparticle size/percent not currently achievable using conventional deposition techniques reported in the literature [15,16,17,18,19]. Such hybrid technologies have the ability to produce nanocomposites and laminar films for modern material needs, such as coatings for solar harvesting applications (i.e., by converting solar energy into thermal power), tribological coatings for energy reduction in vehicles (i.e., new tough coatings with low friction coefficient and density on engine components) and protective coatings in biomedical implants. The a–C:H:Ag and a–C:H:Ti nanocomposite films deposited herein were characterized using X-ray reflectivity (XRR), Raman spectroscopy, Atomic Force Microscopy (AFM) and Scanning/Transmission Electron Microscopy (SEM, TEM) for measuring density, thickness, chemical bonding, composition, roughness and nanoparticle size/shape. The nanoscratch response was quantified using an instrumented indentation platform. The transition metals of silver and titanium were selected as reinforcing nanoparticles due to their very good mechanical compatibility with a–C:H ($E_{\mathrm{Ag}}=70\;\mathrm{GPa}$, $H_{\mathrm{Ag}}=1\;\mathrm{GPa}$ and $E_{\mathrm{Ti}}=115\;\mathrm{GPa}$, $H_{\mathrm{Ti}}=3–5\;\mathrm{GPa}$) and their tendency to reduce residual compressive stresses; an element with carbide-forming abilities, titanium, and an element with non-carbide forming tendency, silver, are investigated for comparison. Beyond their chemical and mechanical characteristics, Ag and Ti have been selected due to their optical characteristics—more precisely, due to the way they interact with light. For example nanoparticulate silver and titanium (in its nitride form), exhibit plasmon-resonance response when interacting with light [20], which translates into an enhanced optical absorption in the UV-visible-near IR range of wavelengths and can be opted in coatings for solar-harvesting applications [21,22]. In fact, their introduction into the amorphous carbon matrix generates a nanocomposite system with additional functionalities. Furthermore, beyond their optical characteristics, silver and titanium have been used in biomedical devices. While the therapeutic window where metallic nanoparticles are bactericidal but retain their biocompatibility characteristics with human cells is still a matter of debate and investigation [23,24,25,26,27,28], this type of nanocomposite systems (a–C:H:Me) could serve as candidate materials for such applications.

## 2. Materials and Methods

### 2.1. Deposition of Nanocomposite a–C:H:Me Films

#### 2.1.1. Ion Beam Source

**Figure 1:**
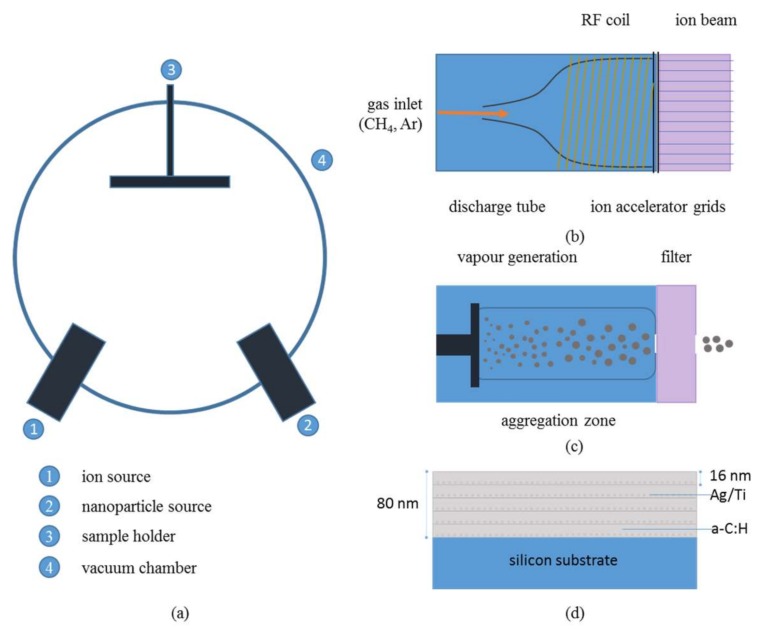
(**a**) Schematic of the hybrid PECVD/PVD system used within this study; Details of (**b**) the ion source and (**c**) the nanoparticle source; (**d**) Schematic of the metal containing hydrogenated amorphous carbon nanocomposite films deposited within this study (a–C:H:Ag and a–C:H:Ti).

Metal-containing hydrogenated amorphous carbon films were deposited using a custom-made hybrid deposition system, which combines plasma enhanced chemical vapor deposition (PECVD) and physical vapor deposition (PVD) technologies. A schematic of the deposition chamber is presented in Figure 1a. PECVD was enabled through radio frequency (RF) ion-beam technology, a diagram of which is presented in Figure 1b. The ion beam source has an external RF antenna that spirals around the plasma tube in the form of a coil. The RF waves emitted by the antenna enter the transparent plasma tube to ionize the gas introduced therein to produce charged ions. The main chamber of the system was pumped down to 10^−8^ mbar (basic pressure) using a roughing and a turbo-molecular pump. The energetic carbon/hydrogen ions generated from this gas-cracking process (methane (CH_4_) was used in this study) were accelerated towards the substrate by a voltage applied on a grid located between the plasma source and the substrate material. The voltage applied on the grid related to the kinetic energy of the ions. The transportation of ions from the source to the substrate occurs in line of sight conditions and a working pressure of approximately 10^−3^ mbar, the exact value of which depends on the total gas flow within the discharge tube. The accelerated ion species were deposited on the substrate material to grow hydrogenated amorphous carbon (a–C:H) films. The ion beam arrived at an incidence angle of 30° to the substrate which was located 22 cm away from the ion beam. In optimizing the density and deposition rate a parametric study on the effects of deposition conditions on the physical characteristics of a–C:H films preceded the deposition of the metal-containing nanocomposite films (a–C:H:Me).

#### 2.1.2. Nanoparticle Source

Metal NPs were generated using NanoGen50 (Mantis Deposition Ltd., Thame, UK). The nanoparticle generator (Figure 1c) utilizes a variant of the PVD method, called Terminated Gas Condensation (TGC); this technology uses magnetron sputtering coupled with a condensation zone that is used to grow metallic NPs. More details on the operating mechanisms of TGC can be found in Ref. [29]. Here, silver or titanium were physically vapored by momentum transfer of argon ions onto the solid target (application of sputtering voltage/power promotes the creation and then acceleration of argon ions onto the solid target). The sputtered atoms of metal target nucleate and grow into larger clusters through collisions in the gas phase. The length of the condensation zone, which can be varied, affects the size distribution of the metallic clusters; longer times spend within the condensation zone result in higher collision in gas phase and thus larger clusters. The size of the metallic NPs have negative charge and can be consequently filtered by a quadrupole (complex of four rods) mass spectrometer MesoQ (Mantis Deposition Ltd., Thame, UK) which is located in series with the condensation zone (see Figure 1c). The quadrupole filter can be used to preselect NPs of specific size (mass) to pass through it and at the same time measure their current (which qualitatively relates to the number of NPs per unit area per time). In order to investigate the control on the particle size, Ag and Ti NPs were deposited on silicon substrates with sub-nanometer roughness of 0.4 nm (measured through AFM). Prior to deposition, the substrates were cleaned with compressed air to remove any possible dust particles or debris from their surfaces. Argon gas flow, sputtering current and condensation distance were set to 60 sccm, 60 mA and 8.5 cm, respectively; the working pressure in the main chamber was in the order of 10^−3^ mbar.

#### 2.1.3. Hybrid Deposition of a–C:H:Me Nanocomposite Films

Nanocomposite films of a–C:H:Ag and a–C:H:Ti were deposited by sequential operation of the PECVD and PVD guns. A pattern of five layers of a–C:H films (each with ~16 nm thickness with a deposition rate of ~4 nm/min) and intermediate depositions of metallic NPs (selected nominal mean particle diameters of 4 nm for Ag and 10 nm for Ti) between each a–C:H layer was implemented. No energetic bias was applied on the substrate while the temperature remained at 25–35 °C. An RF power of 200 W was applied to a CH_4_/Ar mixture of 6.0 sccm/0.5 sccm while the produced carbon ions were accelerated on the substrate material using 150 V grid voltage and a background pressure of 10^−3^ mbar to produce a–C:H layers with a density of 1.7 g/cm^3^ (measured by X-ray reflectivity).

### 2.2. Characterization of Nanoparticles and Films

#### 2.2.1. X-ray Reflectivity

An X-ray diffractometer (Rigaku Ultima IV, Tokyo, Japan) was used to measure the specular X-ray reflectivity of the various deposited films. The diffractometer was equipped with a Cu tube, operated at 40 kV accelerating voltage and 40 mA emission current. The incidence X-ray beam was collimated into a parallel beam with a 0.03 divergence and additionally monochromatized to Cu Ka (λ = 0.15419 nm) by a curved multilayer mirror. Density and thickness values of the thin films were extracted by fitting the respective experimental data to the theoretical reflectivity calculated using Parratt’s formalism [13,30,31].

#### 2.2.2. Raman Spectroscopy

The microstructural details of a–C:H:Me films were probed using Raman spectroscopy. This characterization method was employed in order to (a) access the bond characteristics of the deposited a–C:H films and indirectly link the information with sp^2^/sp^3^ configurations, and hydrogen content (i.e., through *I*_D_/*I*_G_ and full-width at half-maximum of the G-peak, FWHM(G)) [32] and (b) trace the chemical modifications imparted on the nanocomposites, a–C:H:Ag and a–C:H:Ti, through the introduction of metal NPs. Raman data were collected by a confocal LabRAM (HORIBA Jobin Yvon, Kyoto, Japan) equipped with a CCD detector and 1800 grooves/mm grating. It is equipped with an Olympus BX41 microscope (10×, 15×, 40×, 50× and 100×). The 441.1 nm excitation laser beam was provided by a Helium-Cadmium laser (ΚΙΜΜΟΝ ΚΟΗΑ, Fukushima, Japan). The laser power incident on the sample was 3–4 mW and the accumulation time was 15–20 min for each spectrum.

#### 2.2.3. Atomic Force Microscopy

Atomic force microscopy (AFM) was used to quantify the size and number of metallic NPs and also to quantify the roughness of the resulting nanocomposite films. All measurements were performed in semi-contact mode using a scanning probe microscope (Ntegra Prima, NT-MDT, Moscow, Russia) equipped with an NT-MDT cantilever (NSG10) having a mean force constant of 11.8 N/m and a tip nominal radius of 6 nm. For NP size calibration AFM images were 500 nm × 500 nm in size collected with a tip scan rate of 1 Hz and a resolution 512 × 512 points in *x*-*y* direction. The surface topography of the deposited a–C:H and a–C:H:Me films was measured using contact mode and a CSG10 probe having a mean force constant of 0.11 N/m and a nominal tip radius of 6 nm. Images of 3 μm × 3 μm and 256 × 256 data density were collected and subsequently software-analyzed for quantifying the root mean square (RMS) roughness of the deposited a–C:H:Me surfaces.

#### 2.2.4. Nanomechanical Testing

The nanotribological response of the deposited a–C:H:Me films was tested on an instrumented nanoindentation platform (Micro Materials Ltd, Wrexham, UK) using a friction bridge transducer and a conospherical diamond probe with a tip radius of 3.2 μm, as calibrated through elastic indentations on a material with known properties [33]. The three-pass experiment with 1 ramp-load scratch test between 2 topography passes was used for scratch testing all films with 6 repetitions, with a total scratch length of 300 μm, a load applied after 50 μm distance, a scan speed of 2 μm/s and a scratch loading rate of 1.6 mN/s to reach a maximum load of 200 mN. A 50 μm distance between each scratch was used. During all scratch tests friction data was collected that provided access to the friction coefficient.

#### 2.2.5. Transmission/Scanning Electron Microscopy

Selected specimens were studied using TEM (JEM HR-2100, JEOL Ltd., Tokyo, Japan) operated at 200 kV in bright field mode. In studying the shape and size of the generated NPs, several depositions were made directly on formvar/carbon coated 300 mesh Cu TEM grids (Science Services GmbH, München, Germany). Residual scratches from nanomechanical measurements were also studied with SEM (Quanta 200, FEI, Hillsboro, OR, USA) at various magnifications in order to link microstructural characteristics with nanotribological metrics.

#### 2.2.6. Residual Stress Measurements

Residual stresses generated within the film during deposition were estimated using Stoney’s equation [34]:

(1) $$\sigma_{f}=\frac{1}{6}\left(\frac{E_{s}}{1-v_{s}}\right)\frac{t_{s}^{2}}{t_{f}}\left(\frac{1}{R}\right)$$

where *E*_s_ = 170 GPa and *v*_s_ = 0.22 are the silicon substrate modulus of elasticity and Poisson’s ratio, and *R* is the radius of curvature calculated through height and cord measurements of cross-sectional data obtained from three dimensional non-contact optical profilometry surface profiles; height and cord data were recorded with 0.1 nm and 150 nm accuracy, respectively. The validity of Equation (1) was ensured by respecting that the fundamental assumptions on which it was derived persist [35,36]: (i) substrate and film thicknesses are significantly smaller than the plane dimensions, (ii) film thickness (*t*_f_ ≈ 80 nm) is significantly smaller than the substrate thickness (*t*_s_ = 375 μm), (iii) substrate and film are homogeneous, and (iv) residual stresses induce equal bending in both *x*-*y* directions (i.e., spherical curvature and thus the biaxial stress is equal in the whole plate).

## 3. Results and Discussion

### 3.1. Ion Source and a–C:H Deposition Rate

Prior to the deposition of the nanocomposite films a series of pristine a–C:H films were deposited in order to study the effects of ion source characteristics on the deposition rate and physical properties of the films. The resulting thickness and density of the produced films were quantified through X-ray reflectivity measurements. The various depositions performed investigated the parameters that affect the ion beam characteristics and their link with microstructural characteristics of the films so to produce a–C:H with dense packing of carbon atoms and efficient deposition rates. In general, the ion beam process can be divided into three basic steps: (i) creation of ionized species, (ii) acceleration of species, and (iii) deposition and growth of the film; the controlling variables that affect those processes are the RF power, the gas flow, grid voltage, and substrate bias/temperature. For this study, substrate bias and temperature were kept at 0 V and 25 °C respectively.

**Figure 2:**
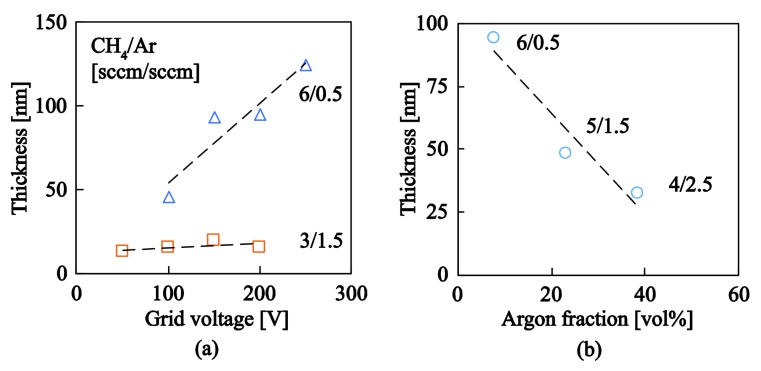
(**a**) Effect of grid voltage on film thickness for two different gas flow rate combinations: CH_4_/Ar = 6.0 sccm/0.5 sccm = 12 and CH_4_/Ar = 3.0 sccm/1.5 sccm = 2; results are for RF power of 200 W; (**b**) Effect of volume fraction on film thickness; results are for RF power of 200 W and grid voltage of 150 V and a constant total gas flow rate in the chamber (CH_4_ + Ar = 6.5 sccm).

In investigating the effect of gas flow rate and grid voltage a series of specimens were deposited while systematically varying these two parameters. Figure 2a shows that the deposition rate increases with grid voltage and the dependency is more pronounced for higher CH_4_/Ar ratios. In the system presented herein the kinetic energy of the accelerated ions is controlled by the voltage applied to the plasma grid of the discharge quartz tube. It is apparent that the increase of grid voltage does not only affect the kinetic energy of the plasma generated ions but also the rate at which they pass through the grid, having as a result an increase in the amount of deposited species and subsequently film thickness. Furthermore, the deposition rate also increases when the CH_4_ concentration in the tube increases. Figure 2b quantifies the effect of argon gas flow on the deposition rate of the a–C:H films. It is apparent that an increase in the relative volume fraction of argon flow relative to the total gas flow in the discharge tube (argon and methane) significantly reduces the resulting film thickness. It should be noted that the total gas flow rate was kept constant (6.5 sccm) such as the pressure within the chamber remained relatively unaffected. The highest thickness among the produced films is observed for Ar/CH_4_ flow rates of 0.5 sccm/6.0 sccm (Ar volume fraction of ~8%) whereas for Ar/CH_4_ flow rates of 2.5 sccm/4.0 sccm (Ar volume fraction of ~38%) the thickness of the deposited films is significantly reduced by −65%. This phenomenon is partly related to the fact that Ar ions generated within the plasma chamber and accelerated towards the substrate promote etching to the surface of the deposited material (it should be noted that argon atoms are significantly heavier than carbon atoms) thus decreasing deposition rate and thickness of a–C:H films. Furthermore, the increase of Ar volume fraction while maintaining a constant gas flow rate within the chamber leads to a gradual reduction in the CH_4_ volume fraction which is the source of carbon ions, and its reduction inevitably leads to a reduction in the deposition rate and thickness of the deposited material. The density values of all films deposited herein vary within 1.52 g/cm^3^–1.80 g/cm^3^.

### 3.2. Ag and Ti Nanoparticles

A series of silver and titanium NP depositions were performed on silicon and TEM grid substrates in order to confirm the ability of the nanoparticle source to generate NPs with precise sizes and calibrate at the same time the deposition rates that were required for the controlled composition of the nanocomposite films. Table 1 shows details of the set of silver nanoparticle samples synthesized using NanoGen50 where the preselected nominal particle size has been varied while retaining a grounded substrate and a constant deposition flux (*K*), defined as the product of NP current (*j*) with deposition time (*t*):

(2) K=j × t

Consequently, for a given ion current (which is experimentally tractable) the deposition flux can be easily controlled by tuning the duration of a given deposition.

**Figure 3:**
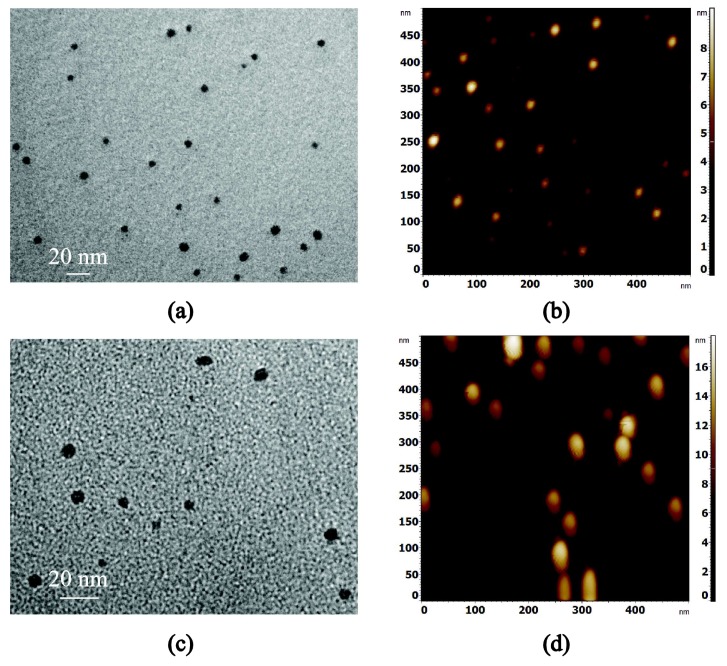
TEM (**a**,**c**) and AFM (**b**,**d**) images of Ag (**a**,**b**) and Ti (**c**,**d**) NPs synthesized using the nanoparticle source. Nominal diameters for Ag and Ti were 4 nm and 10 nm.

**Figure 4:**
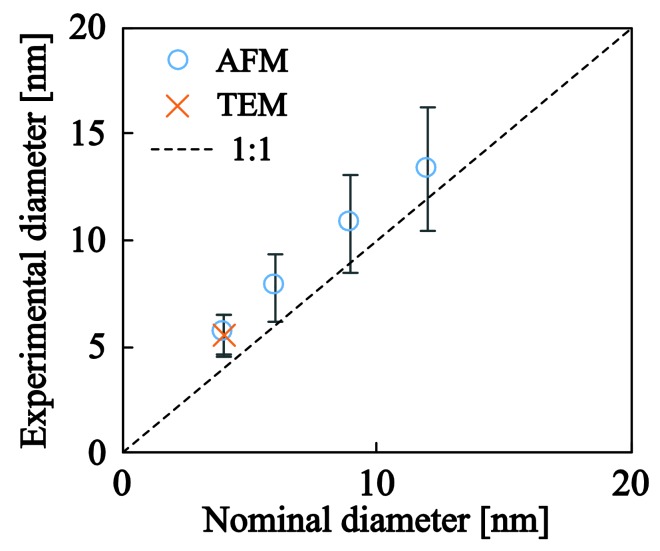
Experimental versus nominal NP diameters for a series of Ag nanoparticle depositions. Experimental diameters were obtained from AFM and TEM images after digital image analysis.

Figure 3 shows TEM (Figure 3a,c) and AFM (Figure 3b,d) images of the generated Ag (Figure 3a,b) and Ti (Figure 3c,d) NPs. The images testify towards the ability of the nanoparticle source to generate a non-agglomerated group of almost spherical NPs with controlled diameters. In order to quantify the sizes of the Ag and Ti NPs the images were digitally analyzed. For all the samples, the size and number of NPs were counted by the threshold method [37,38] which makes the assumption that the NPs are spherical and rest on a substrate with minimal roughness. During the levelling process the particles were excluded from the fit to avoid local distortions of the data. Figure 4 shows the experimentally obtained diameters in comparison with their nominal values for the various Ag NPs synthesized herein. It is evident that the TEM results are in excellent agreement with the AFM data and confirm the ability of NanoGen to deposit NPs with controlled sizes and minimal size distributions. Some minor deviations between the experimental and nominal values can be attributed to the settings of the quadrupole filter which potentially could be optimized for even more refined correspondence between the two.

**Figure 5:**
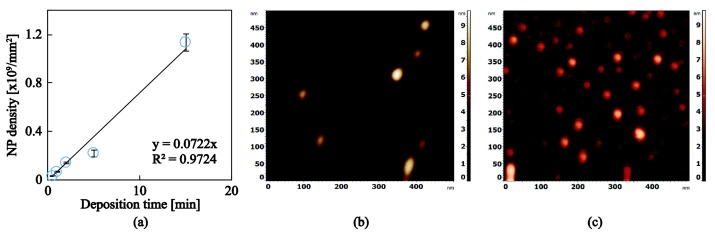
(**a**) Ag nanoparticle density for various deposition times; results relate to the samples shown in Table 2. AFM image of a (**b**) low deposition time and (**c**) high deposition time.

Figure 5a shows the particle density, calculated through AFM images in billions of NPs per square millimeter, as a function of the deposition time. The details of the samples synthesized towards this end are presented in Table 2 and the experiments were performed in order to quantify a measure of the deposition rate that allowed us to generate nanocomposite a–C:H:Me films with controlled compositions. The samples presented in Figure 5a had a nominal size of 4 nm and various deposition times preselected to achieve various coverages/densities. Figure 5b,c show AFM images of low and high NP coverage respectively. The AFM images were analyzed using the threshold routine and the values of NP density are plotted in Figure 5a. As expected, an increase in deposition time increases the number of NPs per mm^2^ and this trend appears to be linear with a resulting deposition rate of 0.0722 × 10^9^ mm^−2^ min^−1^. The same experimental process has been followed for Ti NPs showing an almost identical deposition rate.

### 3.3. a-C:H:Ag and a-C:H:Ti Nanocomposite Films

#### 3.3.1. Microstructural Details and Bonding Characteristics

Details of the nanocomposite films prepared and tested within this study are shown in Table 3. The reported particle size relates to the experimentally obtained values (AFM and TEM analysis) whereas the metal contents were estimated through the calibration curves reported in Figure 4 and Figure 5. The PECVD and PVD guns were operating in alternating turns (5 repetitions each) in order to generate multi-layer films of ~80 nm in thickness and intermediate nanoparticle depositions with concentrations as reported in Table 3. The hydrogenated amorphous carbon matrix used in all nanocomposite films was deposited using an RF power of 200 W, a gas mixture of CH_4_/Ar = 6.0 sccm/0.5 sccm and grid voltage of 150 V, which resulted in a film density of 1.7 g/cm^3^ as quantified through X-ray reflectivity measurements.

**Figure 6:**
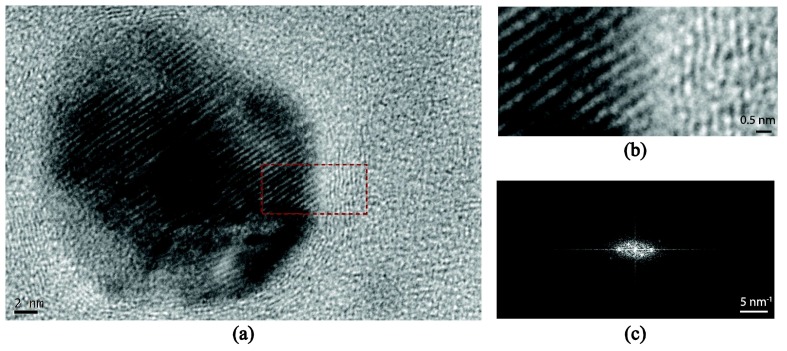
(**a**) High resolution TEM image of a Ti nanoparticle with the surrounding a–C:H matrix; (**b**) A magnified view of the a–C:H/Ti interface showing the transition in crystalline domains; (**c**) 2D FFT of image selection, identifying the two dominant d-spacings.

TEM images suggest that the metallic NPs are crystalline in nature (single crystals in most cases) and retain their original compositions, that is, neither Ag nor Ti NPs chemically react with the surrounding a−C:H matrix. Figure 6 shows a high resolution TEM image of a Ti NP embedded in an a−C:H matrix. A closer investigation at the Ti/a−C:H interphase coupled with a 2-dimensional fast Fourier transform of the cropped section suggests that (a) the nanoparticle exhibits *d*-spacings of 0.234 nm which correspond to the interplanar distance of Ti (002) planes, which translates to the fact that titanium carbide is not formed and (b) the aureole that is formed around the Ti NP shows characteristics of crystallinity with *d*-spacings on the order of 0.172 nm that can be related to the (004) planes of graphite, suggesting that the vicinity to the NPs surface graphitizes, the extend of which can be estimated up to 10 atomic planes (~3 nm). This is consistent with atomistic simulations [39] which reveal that the incorporation of transition metals into the amorphous carbon matrix leads to a process of C–C bond breaking and enables the formation of the lowest energy crystalline carbon state, that of graphite.

**Figure 7:**
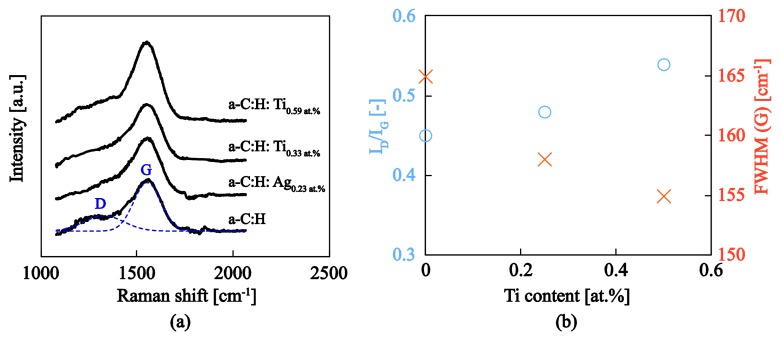
(**a**) Raman spectra for the a–C:H:Me nanocomposite films tested within this study showing also the deconvoluted Raman spectra for a–C:H, fitted by Gaussian curves at D and G carbon band resonant frequencies. (**b**) Extracted *I_D_*/*I_G_* and FWHM(G) as a function of titanium content for the a–C:H:Ti films synthesized herein.

The Raman spectra for a–C:H and a–C:H:Me nanocomposites are presented in Figure 7a. All spectra exhibit the characteristic shape for amorphous carbon; the cumulative response was deconvoluted to the D-band (~$1350{\;\mathrm{cm}}^{-1}$) and G-band (~$1550{\;\mathrm{cm}}^{-1}$) contributions using Gaussian fits. Several important metrics, including the location of the G peak, the intensity ratio of D over G peaks (*I_D_*/*I_G_*) and the full width at half maximum of the G Peak (FWHM (G)) were extracted and the results are shown in Figure 7. In general, the D peak is due to the vibration of sp^2^ rings and the G peak to the resonance of the sp^2^ atoms organized in both rings and chains. Subsequently, the higher the *I_D_*/*I_G_* ratio the higher the sp^2^ clustering within an a–C:H sample.

The intensity ratio of a–C:H yields a value of *I_D_*/*I_G_ =* 0.45. Empirical relations obtained from experimental data on a large collection of data on hydrogenated amorphous carbon films suggest that this intensity ratio is inversely related to both hydrogen content [32] and (indirectly through the Tauc gap) sp^3^ hybridization state [40]. A comparison of this experimentally obtained value with literature data suggests that the pure a–C:H film synthesized within this study consists of a hydrogen content of 20–25 at.% and an ${\mathrm{sp}}^{3}$ content of approximately 50 at.%. An a–C:H film with such characteristics is commonly referred to as diamond-like a–C:H (DLCH) with density values that vary between $1.5\;g/{\mathrm{cm}}^{3}$ to $1.8\;g/{\mathrm{cm}}^{3}$ and high ${\mathrm{sp}}^{3}$ bonds (up to 70 at.%), a significant percentage of which are hydrogenated terminated [1,41]. Indeed, our a–C:H matrix with a density of $1.7\;g/{\mathrm{cm}}^{3}$ (measured through XRR), hydrogen content of ~25 at.% and ${\mathrm{sp}}^{3}$ content of ~50 at.% falls within the DLCH category [32]. It should be noted that the empirical relations utilized herein were obtained with an excitation wavelength of 514 nm, whereas the wavelength utilized in Ref. [32] was 441 nm. Nevertheless, experimental evidence suggest that the effect of excitation wavelength on the *I_D_*/*I_G_* is minimal and therefore the curves obtained with 514 nm excitation are not expected to significantly deviate from the 441 nm results [1,42].

The FWHM(G) probes the structural disorder of the ${\mathrm{sp}}^{2}$ clustering in amorphous carbon material [40,43]. A lower FWHM(G) value denotes an a–C:H with less unstrained ${\mathrm{sp}}^{2}$ clustering, whereas a higher FWHM(G) value suggests a material with an increased disordering in bond lengths and angles for the ${\mathrm{sp}}^{2}$ clusters. The reduction of FWHM(G) with metallic doping (from 165 to 155, see Figure 7b) suggests that the introduction of Ti NPs tends to reduce the structural disorder which is consistent with the graphitization of the surrounding matrix as evidenced in TEM images (Figure 6) and the observed increase in the *I_D_*/*I_G_* ratios and the subsequent reduction of the residual stresses through the relief of strain energy introduced in distorted bond lengths and bond angles. Furthermore, the G peak position shifted slightly to higher wavenumbers, in particular from 1552 cm^−1^ to 1559 cm^−1^ with the incorporation of Ag or Ti metal within a–C:H matrix resulting to higher ${\mathrm{sp}}^{2}$ clustering. This is consistent with the FWHM(G) reduction which also signifies an increase of ${\mathrm{sp}}^{2}$ clustering as it falls following a reverse trajectory from amorphitization for composite samples. Our results are in very good agreement with literature data [17,44,45].

The surface roughness of a–C:H films deposited under controlled conditions onto a flat silicon substrate is measured at 0.4 ± 0.1 nm which is consistent with hydrogenated amorphous carbon values reported in the literature. The introduction of metallic NPs within the a–C:H matrix increases the surface roughness of the a–C:H:Ag and a–C:H:Ti nanocomposite films as presented in Table 3. The increase in roughness for nanocomposites can be attributed to nanoscale protrusions that are generated beyond the NPs as an overlay of a–C:H film is deposited. It is interesting to note that the increase in the roughness for the a–C:H:Ti is more pronounced which is probably related to the higher nanoparticle size used for these nanocomposites in comparison to a−C:H:Ag. Given that surface roughness is one of the parameters that control the interaction of cells with the substrate material they reside [46,47], the ability to manipulate roughness could be exploited for potential use of these materials in biomedical applications.

**Figure 8:**
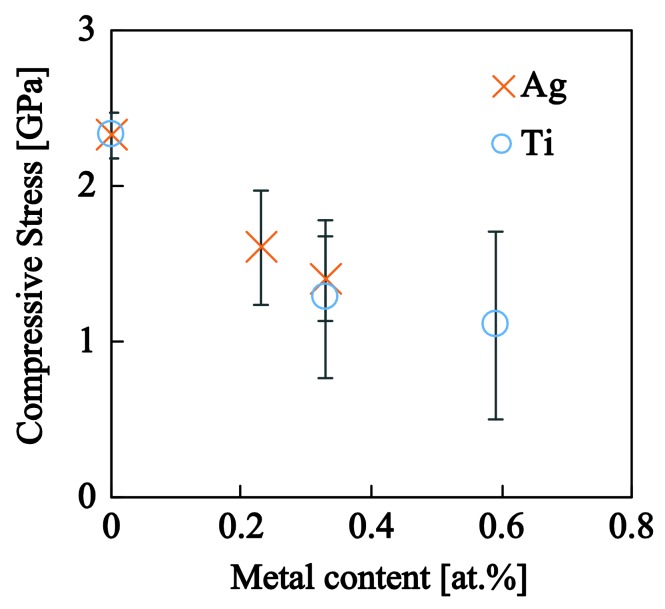
Residual stresses of the nanocomposite films as a function of the metal content.

Residual compressive stresses as calculated through curvature measurements and the Stoney equation are shown in Figure 8. Consistent with literature data, a−C:H films deposited using PECVD contain a significant amount of stresses (~2.3 GPa) that are trapped within the material during the deposition process. The introduction of metallic NPs appears to have a positive effect on the nanocomposite response as a significant proportion of the residual stresses are relaxed: −26% for Ag and −33% for Ti. This is related to the graphitization of the matrix with the introduction of metallic NPs and the release of energy trapped within angular and linear bond-distortions. This observation is also in line with the Raman results presented above. This stress-reduction mechanism increases the stability of the film and enhances the required critical load for film delamination, as it is evidenced below.

#### 3.3.2. Nanotribological Response: Scratch Resistance and Friction Coefficient

**Figure 9:**
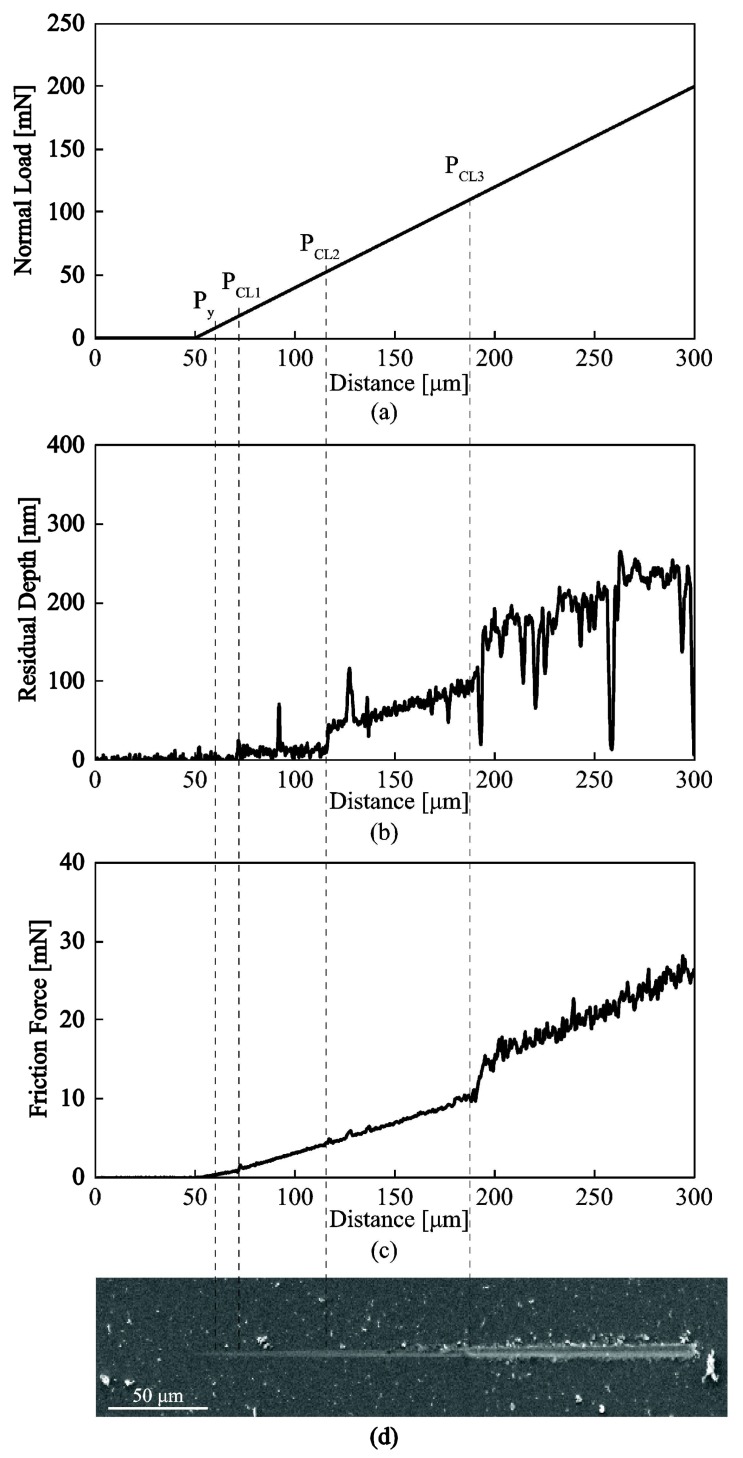
Typical results from a nanoscratch test on a–C:H film. (**a**) Applied load; (**b**) residual depth and (**c**) resulting frictional force as a function of the scratch distance; (**d**) SEM image of the residual nanoscratched imprint.

The tribological performance of all a−C:H and a−C:H:Me nanocomposites deposited within this study was investigated by the nanoscratch method [48,49,50,51] and the extracted critical loads for yielding, cracking, and delamination are reported in Table 4. Figure 9 shows typical data from a three-pass scratch test on a−C:H film, using a conospherical probe. A low load scan before and after the scratch test provided topography information that (a) were used to correct data for initial background tilt and topography and (b) detect film/substrate deformation responses due to applied contact pressures. The key features that are observed in scratch tests are: (a) the load related to the departure from elastic to plastic deformation (denoted as $P_{y}$), (b) the load to initiate film edge cracking (denoted as $P_{\mathrm{CL}1}$ and $P_{\mathrm{CL}2}$), and (c) the load for full film fracture (denoted as $P_{\mathrm{CL}3}$). The load required to initiate plastic deformations can be detected from the deviation of the residual scratch depth from initial topography that is an increase of the residual depth (topography scan during pass 3, Figure 9b). It should be noted that $P_{y}$ is indicative to the substrate initial plastic deformation and not to the film properties itself as evidenced by calculations on the maximum stress location which is well within the substrate material [12,52]. An additional step on the residual scratch depth reveals film crack initiation while any large fluctuations of residual scratch depth relate to film fracture and delamination from the substrate. Furthermore, the friction force evolution with scratch distance (Figure 9c) is an alternative convenient means to detect/confirm $P_{\mathrm{CL}3}$ for delamination fracture. Plastic deformation, cracking and film failure changes were also confirmed microscopically from SEM images. For the pristine a−C:H the average critical loads for yielding, cracking and failure were $P_{y}$ = 5.3 mN, $P_{\mathrm{CL}1}$ = 18.2 mN, $P_{\mathrm{CL}2}$ = 48.0 mN, and $P_{\mathrm{CL}3}$ = 114.8 mN. The extracted critical loads for all films tested herein are summarized in Table 4.

**Figure 10:**
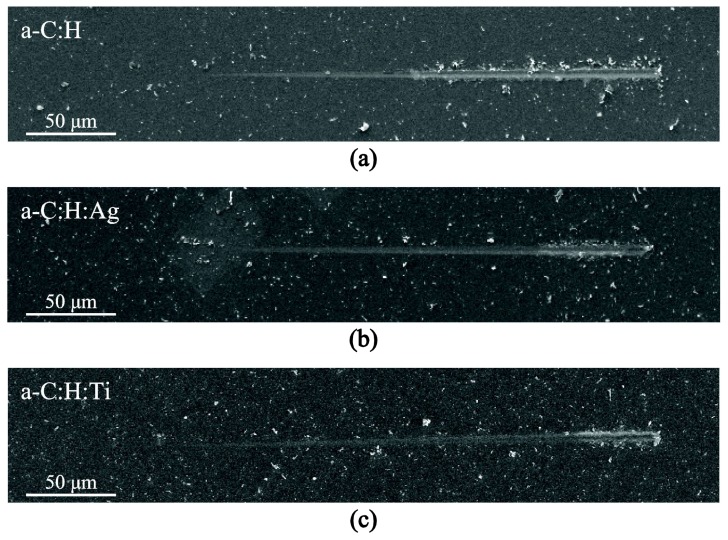
Characteristic residual scratches on (**a**) a−C:H, (**b**) a−C:H:Ag and (**c**) a−C:H:Ti films.

The a−C:H, a−C:H:Ag, and a−C:H:Ti films exhibited similar critical loads for transitioning from elastic to plastic deformations. More precisely, the average $P_{y}$ values for these films were found to be 5.3 mN, 4.5 mN, 5.4 mN, 4.7 mN and 4.9 mN respectively. Beyond that, the average values of the critical loads required for cracking initiation, $P_{\mathrm{CL}1}$, were found to be 18.2 mN, 22.6 mN, 23.5 mN, 18.5 mN and 18.8 mN respectively; for all nanocomposite cases the average values were higher than the value for pristine a−C:H film. Furthermore, the delamination loads found for composite films in all other cases were higher compared to the neat a−C:H film; this can also be clearly observed by comparing the residual imprints. Indicative SEM images of residual imprints on pristine and metal containing nanocomposite films are shown in Figure 10, where it is evident that the initiation of delamination is shifted to higher distances/loads.

**Figure 11:**
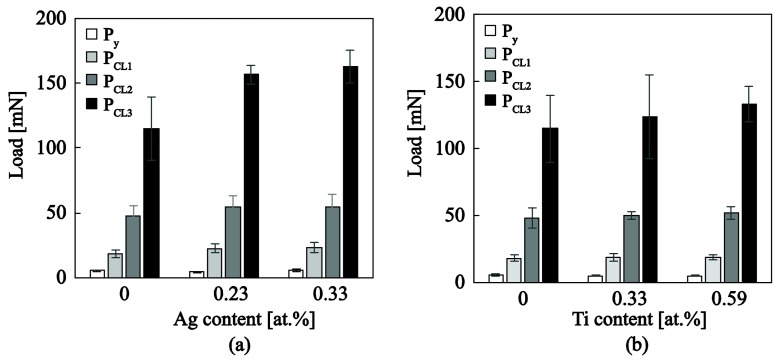
Critical loads for yield, cracking and delamination as quantified through scratch tests for (**a**) a−C:H:Ag and (**b**) a−C:H:Ti.

The enhancement in scratch resistance exhibited by a−C:H:Ag and a−C:H:Ti systems (Figure 11) is in line with bonding characteristics extracted from Raman measurements. The increase of $\left(I_{D}/I_{G}\right)$ ratio and decrease of structural disorder through FWHM(G) value with metal content imply an increase of ${\mathrm{sp}\;}^{2}$ clustering and deviation from amorphitization trajectory and hard materials. It is therefore clear that silver and titanium promote the graphite-like properties and both reduce hardness and increase ductility, toughness and abrasion resistance as well. These conclusions are well supported from experimental and theoretical studies in literature; concerning the increase of ${\mathrm{sp}}^{2}$ clustering with the presence of amount and size of silver [39,45,53], while the decrease of hardness and increase of abrasion resistance is discussed in [12].

**Figure 12:**
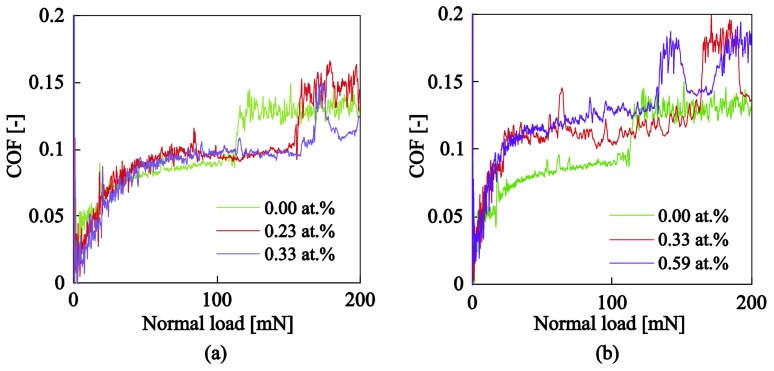
Coefficient of friction as a function of applied load for (**a**) a−C:H:Ag and (**b**) a−C:H:Ti nanocomposite films.

**Figure 13:**
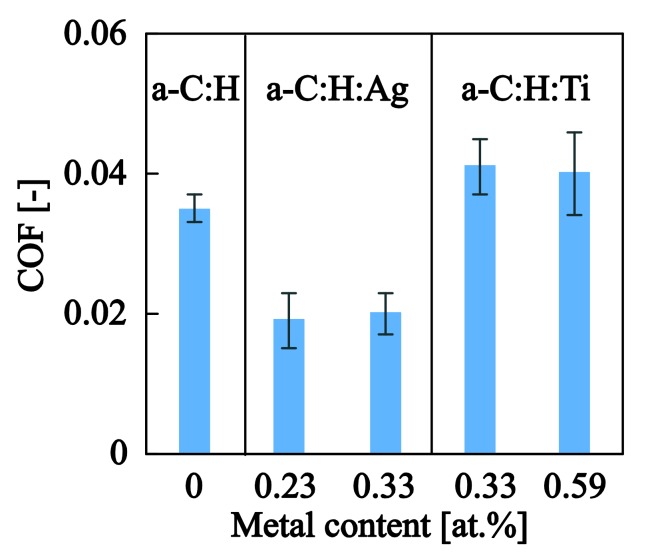
COF values for the a−C:H and a−C:H:Me films deposited in this study. The COF values reported correspond to the average values calculated within the elastic domain ($P<P_{y}$).

For such systems, residual stresses play a pivotal role in the delamination and early fracture during scratching. Residual stresses measured for a−C:H:Me films were found to decrease compared with a−C:H (see Figure 8). The reduction of residual stresses coupled with the chemical, physical and synthesis characteristics of metallic particles appears to be beneficial for the abrasion resistance of the nanocomposite films. For example, the low elastic modulus and hardness of Ag (E≈70 GPa, H≈1 GPa), coupled with its low wettability and inertness with carbon, and good dispersion and geometrical characteristics leads to a reduction of residual stresses, the development of a nanocomposite film which enhances ductility, toughness and scratch resistance to higher values in contrast to the pure a−C:H matrix. Titanium with an elastic modulus of E≈115 GPa and hardness of H≈3−5 GPa exhibits similar enhancements on the tribological response of the nanocomposite films. The lower improvements can be attributed to the higher roughness (probably caused by the higher nanoparticle size, 11.3 nm compared to 5.6 nm for silver) that amplifies the ploughing contribution of roughness and subsequently the frictional resistance (see Figure 12 and Figure 13).

The coefficient of friction (COF) is another property of great significance which controls the tribological characteristics of these type of films. Figure 12a,b shows the evolution of COF with the applied load during the scratch test for a−C:H:Ag and a−C:H:Ti respectively. For low loads the contact pressure is below the yield limit of either the film or the substrate and the COF is very low (~0.02–0.04). As the contact pressure increases COF increases in a non-linear fashion with the applied load, subsequently reaching constant values until the film fails which can be noted with a sudden increase in the COF vs. applied load response. The increase of COF with the applied load relates to the plasticity and ploughing effect of diamond probe within the a–C:H film [54]. As COF increases the stress beneath the contacting probe becomes more significant with the possibility of failure to follow the probe [55]. Such a response is not directly detectable in the COF vs. applied load evolution but can be observed in the residual surface profiles through probe line scans or SEM images (see Figure 9).

The average COF values calculated within the elastic contact regime ($P<P_{y}$) for all films tested herein are shown in Figure 13. The COF value for pure a−C:H is 0.035 ± 0.002 which is in agreement with values reported in the literature for similar material systems and testing conditions [56]. The very low COF values have been attributed to the formation of a tribolayer between the probe and the film, which acts as a solid lubricant that suppresses both COF and wear. The introduction of silver into the system tends to lower the COF into even smaller values (~0.020). The enhancement of lubrication in the silver-doped amorphous carbon systems has been reported in [57,58] and can be attributed to the solid lubricant properties induced by the soft silver NPs [45,59]. The introduction of Ti NPs into a−C:H tends to slightly increase the COF (even though the increase is within the standard deviation of the experimental results) which might be attributed to the increased roughness generated by the larger size of Ti NPs and the increased elasticity/hardness of the Ti NPs which might delay the yielding of a−C:H and the generation of the transfer layer. An increased COF for surfaces with higher roughness has also been documented by Erdemir et al. [60] and has been attributed to the more extensive ploughing contribution that existed in such surfaces.

## 4. Conclusions

A novel hybrid (PECVD/PVD) deposition system is presented that can deliver nanocomposite a−C:H:Me films with dispersed NPs of controlled size and content. The matrix characteristics can be tailored by controlling the gas flow within the discharge tube, the RF power and the grid voltage. The nanoparticle source can deliver non-agglomerated spherical NPs whereas the quadruple filter (MesoQ) can narrow the particle size distribution and pre-select monodisperse particles with nanometer accuracy. AFM and TEM results testified towards the ability of particle size monitoring and were also used to calibrate the deposition rate for controlled nanocomposite film compositions. The PECVD system delivered an a−C:H matrix with 20–25% hydrogen content and ${\mathrm{sp}}^{3}\;$content of about 50% as estimated through Raman spectroscopy. Hydrogenated amorphous carbon films with embedded Ag or Ti NPs were deposited and exhibited very low coefficients of frictions (<0.05) and enhanced nanoscratch resistance (up to +50%). This improved nanotribological response can be traced to the reduced residual stresses, and the higher matrix ductility caused by the graphitization of the a−C:H through the release of strain energy and the nanocomposite morphology that increases the toughness of the material. These improved material systems retain their nanometer scale roughness and could be potentially exploited for biomedical or other protective applications.

## Figures and Tables

**Table 1:** Deposition details for a series of silver nanoparticle specimens with nominal diameters in the 4 to 12 nm range. Argon flow, magnetron position, and NP current where set at 60 sccm, 8.5 cm, and 60 mA respectively. The substrate was grounded and the size was selected using the MesoQ filter.

Samples	Working Pressure (mbar)	Nominal Size (nm)	Deposition Time (s)	J (nA)	*K* (nA·s)
**Ag_12nm_**	4.9 × 10^−3^	12	60	0.03	1.8
**Ag_9nm_**	4.8 × 10^−3^	9	18	0.10	1.8
**Ag_6nm_**	4.8 × 10^−3^	6	11	0.17	1.8
**Ag_4nm_**	4.8 × 10^−3^	4	45	0.04	1.8

**Table 2:** Deposition conditions for 4 nm nominal diameter Ag NPs grown at various durations and 0 V substrate bias. Argon flow, magnetron position, and NP current were set at 60 sccm, 8.5 cm and 60 mA.

Samples	Working Pressure (mbar)	Nominal Size (nm)	Deposition Time (s)	*J* (nA)	*K* (nA·s)
**Ag_30s_**	4.7 × 10^−3^	4	30	0.3	9
**Ag_60s_**	4.7 × 10^−3^	4	60	0.3	18
**Ag_120s_**	4.7 × 10^−3^	4	120	0.3	36
**Ag_300s_**	4.7 × 10^−3^	4	300	0.3	90
**Ag_900s_**	4.7 × 10^−3^	4	900	0.3	270

**Table 3:** Deposition details of the nanocomposite films grown in this study and the resulting surface roughness as quantified through AFM. PVD conditions: Argon flow, magnetron position, and sputtering current were set at 60 sccm, 8.5 cm and 60 mA. PECVD conditions: 6 sccm/0.5 sccm (CH_4_/Ar), 200W RF power and 150 V grid current. The Me content is calculated through the calibration curve presented in Figure 5 and the measured NP size and a–C:H deposition rate.

Material	NP Size (nm)	Me Content (at.%)	RMS Roughness (nm)
a−C:H	-	0	0.4 ± 0.1
$a-C:H:{\mathrm{Ag}}_{0.23\mathrm{at}.\;\%}$	5.6	0.23	1.8 ± 1.0
$a-C:H:{\mathrm{Ag}}_{0.33\mathrm{at}.\%}$	5.6	0.33	2.0 ± 0.6
$a-C:H:{\mathrm{Ti}}_{0.33\mathrm{at}.\%}$	11.3	0.33	5.3 ± 0.9
$a-C:H:{\mathrm{Ti}}_{0.56\mathrm{at}.\%}$	11.3	0.59	4.8 ± 0.2

**Table 4:** Summary of tribomechanical metrics extracted from nanoscratch tests.

Samples	$P_{y}$ (mN)	$P_{\mathrm{CL}1}$ (mN)	$P_{\mathrm{CL}2}$ (mN)	$P_{\mathrm{CL}3}$ (mN)	COF (-)
a–C:H	5.3 ± 0.6	18.2 ± 2.7	48.0 ± 7.7	114.8 ± 24.7	0.035 ± 0.002
a–C:H:Ag_0.23at.%_	4.5 ± 0.3	22.6 ± 3.7	54.2 ± 9.1	156.6 ± 7.5	0.019 ± 0.004
a–C:H:Ag_0.33at.%_	5.4 ± 0.7	23.5 ± 3.8	54.7 ± 9.3	162.8 ± 12.5	0.020 ± 0.003
a–C:H:Ti_0.33at.%_	4.7 ± 0.6	18.5 ± 2.9	49.8 ± 2.9	123.8 ± 31.5	0.041 ± 0.004
a–C:H:Ti_0.59at.%_	4.9 ± 0.4	18.8 ± 1.7	52.0 ± 5.0	133.0±13.1	0.040 ± 0.006
